# Early Risk Stratification for In-Hospital Mortality in Patients Undergoing Emergency Surgery for Perforated Peptic Ulcer: A 27-Year Retrospective Cohort Study

**DOI:** 10.3390/jcm15218553

**Published:** 2026-11-03

**Authors:** Egemen Cicek, Cem Deniz Ayıkol

**Affiliations:** 1 Department of General Surgery, Division of Gastroenterological Surgery, Faculty of Medicine, İnönü University, 44280 Malatya, Türkiye; 2 Department of General Surgery, Faculty of Medicine, İnönü University, 44280 Malatya, Türkiye; drcemdenizayikol@gmail.com

**Keywords:** perforated peptic ulcer, emergency surgery, mortality, ASA physical status classification, blood urea nitrogen, risk stratification

## Abstract

**Background/Objectives**: Perforated peptic ulcer (PPU) is a serious clinical condition requiring emergency surgery and continues to carry a significant risk of mortality. This study evaluated factors associated with in-hospital mortality and the discriminatory performance of admission parameters. **Methods**: We retrospectively studied 385 adults who underwent emergency surgery for PPU between January 1999 and December 2025. Survivors and non-survivors were compared using nonparametric tests for continuous variables. A multivariable logistic regression model included 265 complete cases (40 deaths), and selected admission variables were evaluated using receiver operating characteristic (ROC) analysis. **Results**: In-hospital mortality occurred in 49 patients (12.7%). Non-survivors were older (median 76.50 vs. 55.00 years; *p* < 0.001) and had lower hemoglobin (12.30 vs. 14.90; *p* < 0.001) and higher BUN levels (36.00 vs. 21.00; *p* < 0.001). Time from symptom onset to admission did not differ significantly between groups (median 24 vs. 16 h; *p* = 0.471). After adjustment, ASA class remained associated with mortality (adjusted OR 5.404; 95% CI 2.640–11.064; *p* < 0.001), whereas BUN did not (adjusted OR 1.010; 95% CI 0.988–1.032; *p* = 0.378). ROC AUC values were 0.801 for ASA, 0.788 for age, and 0.749 for BUN. **Conclusions**: ASA class was independently associated with in-hospital mortality in this cohort. Although BUN showed discrimination in ROC analysis, its adjusted association was not statistically significant. Findings and exploratory cut-offs require external validation.

## 1. Introduction

Peptic ulcer perforation (PPU) represents an acute and potentially life-threatening complication of peptic ulcer disease, for which prompt surgical intervention is frequently required [1,2,3]. Despite substantial advances in diagnostic strategies, antimicrobial therapy, perioperative resuscitation, critical care, and surgical management, morbidity and mortality following PPU remain clinically important [1,2,3,4]. Several clinical characteristics have been associated with unfavorable outcomes in PPU, including older age, a higher burden of comorbid disease, impaired physiological status at presentation, renal dysfunction, and delayed treatment [4,5].

Early assessment of mortality risk in patients with PPU may help guide perioperative management from the time of presentation. Identifying high-risk patients can inform the intensity of resuscitation, the level of postoperative surveillance required, and the appropriate use of critical care resources [3,6].

Mortality risk in PPU has been evaluated using several prognostic tools, most notably the Boey score, Peptic Ulcer Perforation (PULP) score, Mannheim Peritonitis Index (MPI), and American Society of Anesthesiologists (ASA) physical status classification [6,7,8,9,10]. The Boey score is based on three clinical features: severe comorbidity, shock before surgery, and a perforation duration exceeding 24 h [7]. In contrast, the PULP score incorporates a wider range of variables, including age, comorbidity, ASA class, shock, treatment delay, and renal function [8].

In the original PULP study, the PULP score demonstrated better discrimination for mortality than either ASA or Boey [8]. However, subsequent evaluations have reported the inconsistent performance of these prognostic tools across different patient populations [9,10]. Furthermore, a recent systematic review and meta-analysis of 36 studies found no significant difference in mortality discrimination between the PULP and Boey scores [11].

Given the variable performance of established scoring systems, routinely collected clinical and laboratory data may offer additional information for early risk assessment. Previous studies have associated mortality in PPU with age, physiological impairment, renal function, hematological parameters, and delays in presentation, although the strength and discriminatory value of these associations may vary across patient populations [4,5,9,12].

Rather than developing an additional complex prognostic model, this study focused on routinely available parameters that can be obtained without extra testing, cost, or delay. We aimed primarily to determine which clinical and laboratory factors were associated with in-hospital mortality in patients undergoing emergency surgery for PPU at a tertiary care center during a 27-year period. We also evaluated the discriminatory performance of selected admission parameters by ROC analysis to examine their potential contribution to early mortality risk stratification. We further sought to assess these associations after adjustment for relevant demographic and clinical characteristics and calendar year.

## 2. Materials and Methods

### 2.1. Study Design and Patient Population

Consecutive adult patients who underwent surgical treatment for PPU at the Department of General Surgery, Inonu University Faculty of Medicine, between January 1999 and December 2025 were retrospectively identified from institutional records. The study was conducted in accordance with the principles of the Declaration of Helsinki and was approved by the Inonu University Health Sciences Non-Interventional Clinical Research Ethics Committee (Decision No. 2026/10055; approval date: 5 May 2026).

Patients were eligible for inclusion if they were aged ≥18 years and had a gastric or duodenal peptic ulcer perforation confirmed intraoperatively. Exclusion criteria comprised gastrointestinal perforation secondary to trauma or malignancy, postoperative gastrointestinal leakage, and medical records with insufficient data to evaluate the study variables.

### 2.2. Data Collection

Study data were retrospectively obtained from institutional medical records and electronic health information systems. Collected variables encompassed demographic characteristics, comorbidities, clinical findings at presentation, laboratory measurements, perioperative data, postoperative complications, and clinical outcomes.

Demographic data included age and sex, while recorded comorbidities comprised chronic obstructive pulmonary disease (COPD), hypertension, coronary artery disease (CAD), diabetes mellitus (DM), chronic kidney disease (CKD), and malignancy. Clinical assessment included the time from symptom onset to hospital admission, systolic and diastolic blood pressure, heart rate, ASA physical status classification [13], perforation site and diameter, radiographic detection of free intraperitoneal air, and histories of previous surgery and peptic ulcer disease.

Laboratory data consisted of white blood cell count, hemoglobin, blood urea nitrogen (BUN), creatinine, C-reactive protein (CRP), procalcitonin (PCT), and serum albumin. Operative and postoperative data covered the surgical approach, duration of anesthesia, drain and nasogastric tube use and removal, time to initiation of oral intake, length of hospital stay, and requirement for ventilatory support. Postoperative events evaluated as complications included pneumonia, atelectasis, surgical site infection, evisceration, ileus, repair leakage, and reoperation. Procalcitonin testing was introduced at our institution in 2016. Serum PCT concentrations were measured using the electrochemiluminescence immunoassay (ECLIA) method on a Roche cobas e-series analyzer. PCT measurements were available for 122 of 385 patients (31.7%). Missing PCT values were not imputed, and analyses involving PCT were restricted to patients with available measurements.

Surgical management. Open surgery was performed in 370 patients (96.1%), laparoscopic surgery in 12 patients (3.1%), and the surgical approach was undocumented in three patients (0.8%). Procedures included Graham patch repair, modified Graham patch repair, and gastrectomy.

### 2.3. Outcomes

The primary outcome was in-hospital mortality. Patients were classified as survivors or non-survivors on the basis of their status at hospital discharge, and the two groups were compared with respect to demographic, clinical, laboratory, and perioperative characteristics.

Secondary analyses examined factors independently associated with in-hospital mortality and evaluated the discriminatory ability of selected clinical and laboratory parameters using receiver operating characteristic (ROC) curve analysis.

### 2.4. Statistical Analysis

All statistical analyses were performed with IBM SPSS Statistics version 26.0 (IBM Corp., Armonk, NY, USA). The normality of continuous variables was assessed using the Kolmogorov–Smirnov test. As some variables did not follow a normal distribution, continuous variables were expressed as median (25th–75th percentile), while categorical variables were presented as frequencies and percentages [*n* (%)]. Comparisons between survivors and non-survivors were performed using the Mann–Whitney U test for continuous variables and Pearson’s chi-square test for categorical variables.

Multivariable binary logistic regression analysis was performed to identify factors associated with in-hospital mortality. Candidate variables were selected based on clinical relevance and data availability. ASA class, sex, age, chronic obstructive pulmonary disease, hypertension, coronary artery disease, diabetes mellitus, chronic kidney disease, malignancy, systolic and diastolic blood pressure, blood urea nitrogen (BUN), and calendar year were simultaneously entered into the model using the Enter method. ASA class was treated as an ordinal numerical variable, with the odds ratio representing the change associated with each one-class increase. Multicollinearity was assessed using variance inflation factor (VIF) values. Adjusted odds ratios (ORs) and 95% confidence intervals (CIs) were reported.

Receiver operating characteristic (ROC) curve analysis was used to evaluate how well selected clinical and laboratory variables discriminated in-hospital mortality. For each parameter, the area under the curve (AUC) with its standard error and 95% confidence interval was determined, together with sensitivity and specificity. The optimal threshold for each variable was selected as the value yielding the maximum Youden index (J = sensitivity + specificity − 1). Cut-off values were considered exploratory estimates derived from the study population.

Missing data were handled using a complete-case approach, without imputation. Of the 385 patients included in the study, 265 (68.8%) had complete data for all variables included in the multivariable logistic regression model, including 40 patients who died during hospitalization. The numbers of available observations for individual ROC analyses were 372 for age, 296 for ASA classification, 343 for BUN, 120 for CRP, and 349 for hemoglobin. Accordingly, the proportions of missing observations were 3.4%, 23.1%, 10.9%, 68.8%, and 9.4%, respectively. Differences in data availability across variables should be considered when interpreting the findings. All tests were two-sided, with *p* <0.05 considered statistically significant.

Use of Generative Artificial Intelligence: Generative AI (ChatGPT (OpenAI, GPT-6)) was used solely for English language editing and manuscript organization; it was not used for data generation, statistical analysis, or interpretation of scientific results. 

## 3. Results

### 3.1. Patient Population and Mortality

A total of 385 patients who underwent emergency surgery for perforated peptic ulcer were included in the study. Of these, 336 patients (87.3%) survived, while 49 patients (12.7%) experienced in-hospital mortality.

As shown in Table 1, patients who died were significantly older than those who survived (*p* < 0.001). A statistically significant difference was also observed in sex distribution between the groups (*p* < 0.001), with a higher proportion of female patients in the mortality group (36.7%) than in the survivor group (16.1%).

Regarding comorbidities, the prevalence of chronic obstructive pulmonary disease (18.4% vs. 4.2%), hypertension (36.7% vs. 11.0%), coronary artery disease (24.5% vs. 4.8%), diabetes mellitus (14.3% vs. 3.6%), chronic kidney disease (16.3% vs. 2.4%), and malignancy (22.4% vs. 4.5%) was significantly higher among patients who died than among survivors (*p* = 0.001 for diabetes mellitus; *p* < 0.001 for all other comorbidities).

### 3.2. Clinical and Laboratory Findings

Table 2 presents the comparison of clinical and laboratory parameters according to in-hospital mortality status. The median time from symptom onset to hospital admission was 24 h in patients who died and 16 h in survivors; however, the difference between the groups was not statistically significant (*p* = 0.471). Systolic blood pressure was significantly lower in patients who died than in survivors (120 vs. 125 mmHg; *p* = 0.049), whereas no significant differences were observed in diastolic blood pressure (*p* = 0.138) or heart rate (*p* = 0.998).

Regarding laboratory parameters, white blood cell count (10.60 vs. 13.40; *p* = 0.024) and hemoglobin levels (12.30 vs. 14.90; *p* < 0.001) were significantly lower in patients who died compared with survivors. In contrast, blood urea nitrogen (BUN) (36.00 vs. 21.00; *p* < 0.001), creatinine (1.68 vs. 0.96; *p* < 0.001), and C-reactive protein (CRP) levels (11.70 vs. 3.95; *p* = 0.037) were significantly higher in patients who died. Although the median procalcitonin level was higher in the mortality group, the difference between the groups was not statistically significant (*p* = 0.202).

Furthermore, the perforation diameter was significantly larger in patients who died than in survivors (1.00 vs. 0.50 cm; *p* < 0.001).

### 3.3. Perioperative and Postoperative Findings

Table 3 presents the comparison of perioperative and postoperative characteristics according to in-hospital mortality status. Postoperative albumin levels were significantly lower in patients who died than in survivors (2.00 vs. 2.80; *p* < 0.001). Anesthesia duration was significantly longer in patients who died (120 vs. 90 min; *p* < 0.001), and a statistically significant difference was also observed between the groups in the time to drain removal (*p* < 0.001). The time to nasogastric tube removal (6 vs. 3 days; *p* < 0.001), time to initiation of oral feeding (7 vs. 4 days; *p* < 0.001), and duration of mechanical ventilation (5.50 vs. 1.00 days; *p* < 0.001) were significantly longer in patients who died than in survivors. Although the median length of hospital stay was 7 days in patients who died and 6 days in survivors, the difference was not statistically significant (*p* = 0.482).

Regarding postoperative complications, the proportion of patients requiring mechanical ventilation was significantly higher among those who died (77.6%) than among survivors (8.6%; *p* < 0.001). Similarly, the rates of atelectasis (32.7% vs. 7.4%; *p* < 0.001), pneumonia (38.8% vs. 6.0%; *p* < 0.001), perforation repair site leakage (10.2% vs. 2.1%; *p* = 0.002), and reoperation (6.1% vs. 1.2%; *p* = 0.016) were significantly higher in patients who died. In contrast, no statistically significant differences were observed between the groups in surgical site infection (*p* = 0.300), evisceration (*p* = 0.978), or postoperative ileus (*p* = 0.582).

### 3.4. Factors Independently Associated with Mortality

A total of 265 patients, including 40 patients who died during hospitalization, were included in the multivariable binary logistic regression analysis using the Enter method. After adjustment for the other covariates, ASA class remained significantly associated with in-hospital mortality (adjusted OR: 5.404; 95% CI: 2.640–11.064; *p* < 0.001). BUN was not independently associated with mortality in the adjusted model (adjusted OR: 1.010; 95% CI: 0.988–1.032; *p* = 0.378). Sex, age, comorbidities, systolic and diastolic blood pressure, and calendar year were also not statistically significant (all *p* > 0.05). VIF values ranged from 1.113 to 2.088 (Table 4).

In a secondary exploratory logistic regression model including ASA class and postoperative day 1 albumin, higher ASA class was associated with increased odds of in-hospital mortality (OR = 5.032, 95% CI: 2.454–10.319; *p* <0.001), whereas higher postoperative albumin was associated with lower odds of mortality (OR = 0.444, 95% CI: 0.207–0.952; *p* = 0.037) (Table 5). This postoperative model was evaluated separately from the admission-based multivariable model in Table 4.

### 3.5. ROC Analysis

**Figure 1:**
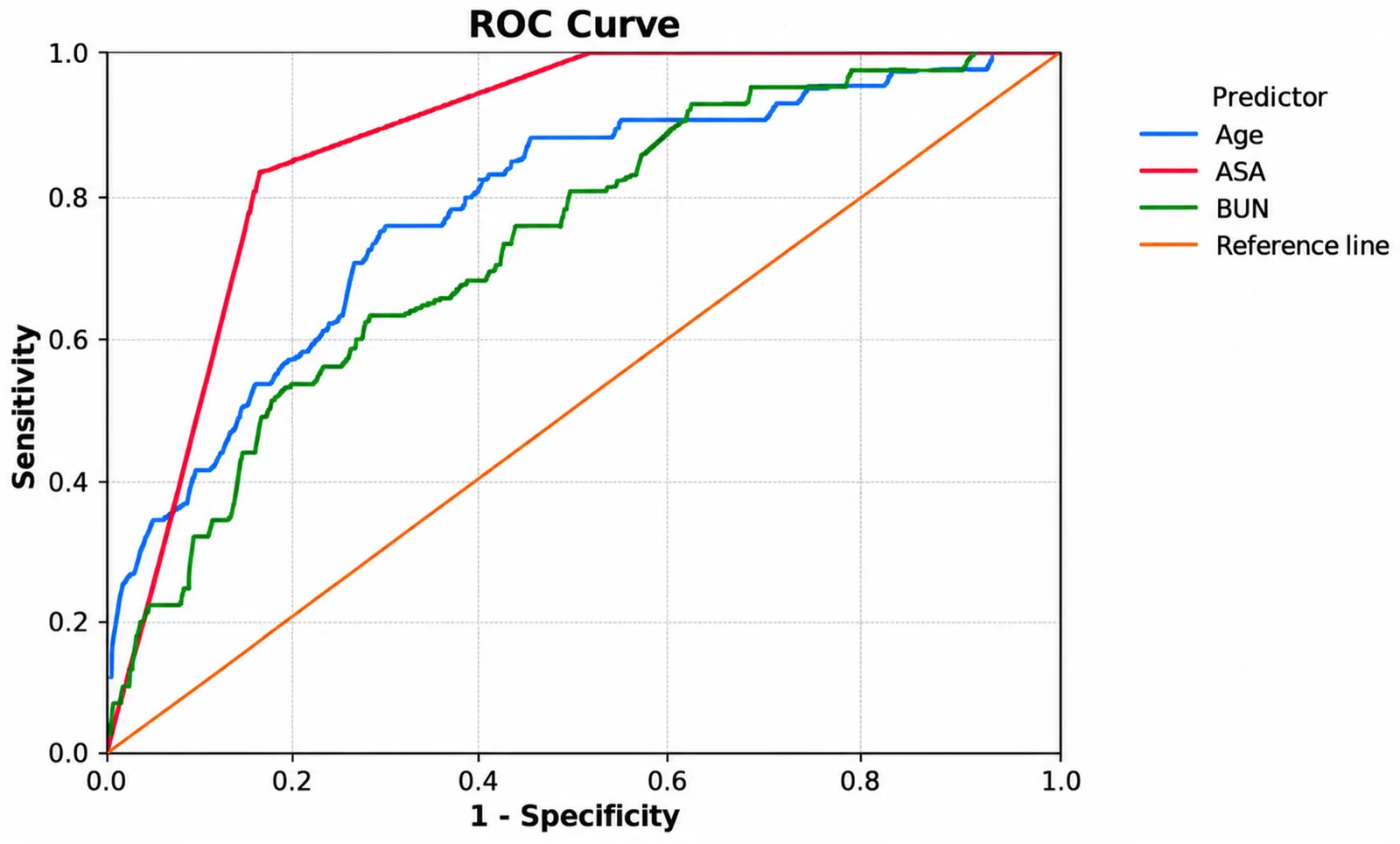
Receiver operating characteristic (ROC) curves for age, ASA classification, and blood urea nitrogen (BUN) in predicting in-hospital mortality.

In the updated ROC analysis, ASA class (*n* = 296) showed an AUC of 0.801 (95% CI: 0.661–0.941; *p* = 0.002), with 92.3% sensitivity and 70.4% specificity at a cut-off of 2.5. Age (*n* = 372) showed an AUC of 0.788 (95% CI: 0.633–0.942; *p* = 0.004), with 76.9% sensitivity and 77.8% specificity at a cut-off of 69.5 years. BUN (*n* = 343) showed an AUC of 0.749 (95% CI: 0.581–0.917; *p* = 0.012), with 53.8% sensitivity and 92.6% specificity at a cut-off of 36.925. CRP (*n* = 120) showed an AUC of 0.707 (95% CI: 0.548–0.865; *p* = 0.036), with 100% sensitivity and 44.4% specificity at a cut-off of 2.775 (Table 6). The ROC curves for age, ASA class, and BUN are presented in Figure 1.

**Figure 2:**
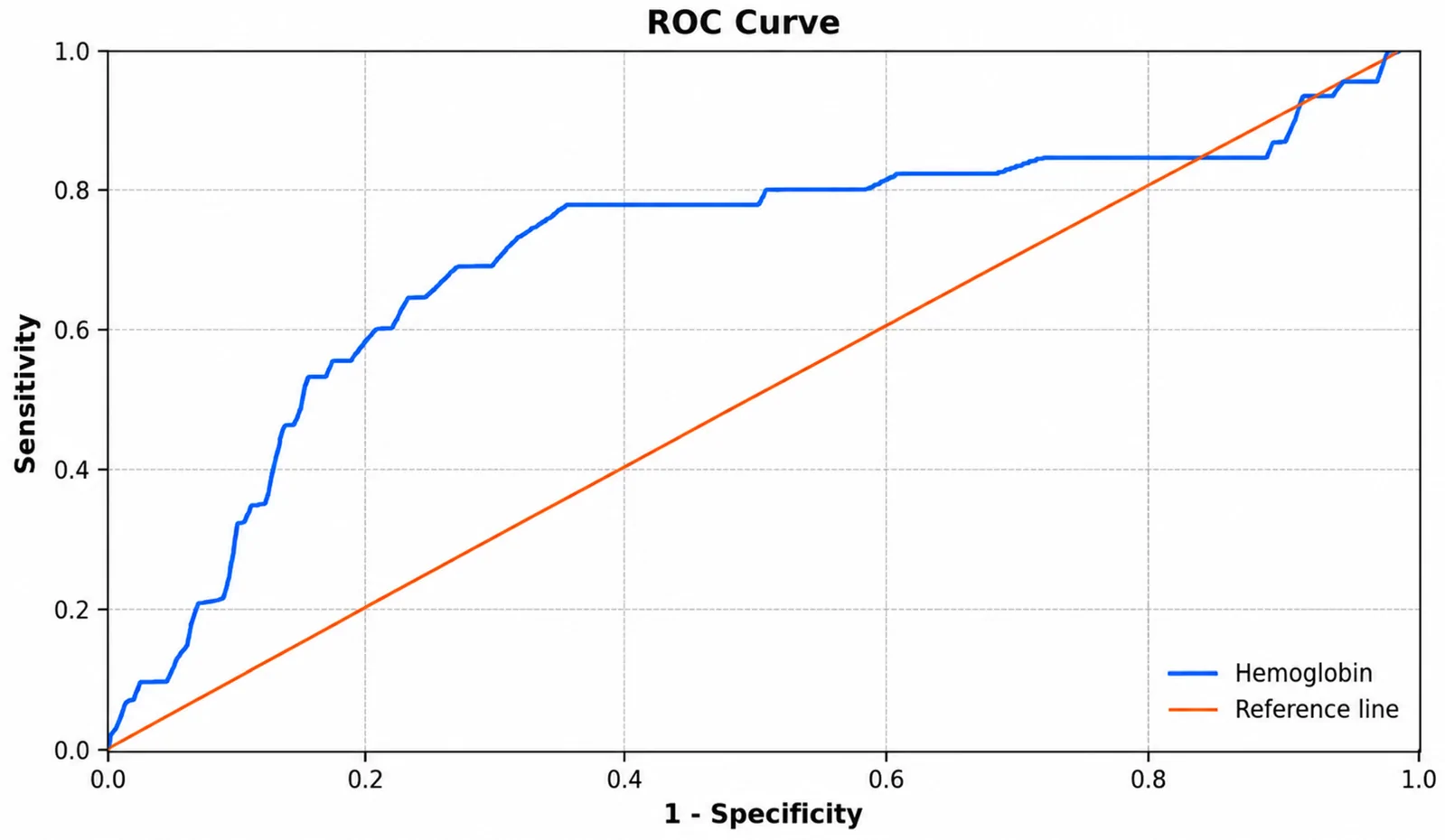
Receiver operating characteristic (ROC) curve for hemoglobin in predicting in-hospital mortality.

Hemoglobin was also evaluated in the ROC analysis (*n* = 349; AUC = 0.685, 95% CI: 0.582–0.788; Table 7 and Figure 2). Postoperative variables were not included in the admission-based ROC assessment.

## 4. Discussion

In this 27-year cohort of 385 patients undergoing emergency surgery for PPU, in-hospital mortality was 12.7%. ASA class remained associated with mortality after adjustment for demographic characteristics, comorbidities, blood pressure, BUN, and calendar year. BUN was higher among non-survivors in the unadjusted comparison and demonstrated discriminatory ability in ROC analysis, but was not independently associated with mortality in the adjusted model. Non-survivors were older and had lower hemoglobin levels and larger perforation diameters. Although median symptom-to-admission time was numerically longer in non-survivors, this difference was not statistically significant (*p* = 0.471).

Mortality after PPU appears to reflect the combined effects of baseline patient characteristics and the severity of the acute clinical condition. Previous studies have associated adverse outcomes with advanced age, comorbidity, physiological derangement at presentation, impaired renal function, intraperitoneal contamination, and treatment delay [4,5]. Although advances in surgical care, resuscitation, antimicrobial therapy, and intensive care have improved the management of PPU, a substantial mortality burden persists [1,2,3,4,5]. The in-hospital mortality rate of 12.7% observed in our cohort is consistent with the range described in previous studies [1,2,3,4,5].

Recent studies have reported considerable variation in mortality following perforated peptic ulcer. Yalcin et al. reported a postoperative mortality rate of 4.7% among 318 surgically treated patients, whereas Pouroushaninia et al. observed a mortality rate of 18.6% in a cohort of 102 patients [14,15]. In a multicenter Japanese study involving 703 patients, Hoshi et al. reported an overall in-hospital mortality rate of approximately 5%; however, their cohort included patients managed nonoperatively [16]. The 12.7% in-hospital mortality observed in our exclusively surgical cohort falls within this reported range. Nevertheless, differences in patient selection, treatment strategies, mortality definitions, and follow-up periods limit direct comparisons between studies.

ASA physical status was associated with in-hospital mortality in the adjusted model, with each one-class increase corresponding to an adjusted OR of 5.404 (95% CI: 2.640–11.064; *p* < 0.001). ASA also showed an AUC of 0.801, and the cohort-derived cut-off of 2.5 provided 92.3% sensitivity and 70.4% specificity. Given the limited number of outcome events relative to model covariates, this association should be interpreted cautiously.

The prognostic relevance of ASA in PPU has also been reported in previous studies [5,6,9,10]. Thorsen et al. found AUC values of 0.79 for both ASA and PULP and 0.75 for the Boey score [9]. The AUC of 0.801 obtained for ASA in our cohort was similar to that reported for ASA in their study.

More recent evidence further supports the importance of preoperative physiological status in patients undergoing surgery for PPU. In a retrospective study of 318 patients, Yalcin et al. found that advanced age, preoperative shock, renal failure, treatment delay, and intraperitoneal contamination were important adverse prognostic factors [14]. These findings are consistent with the broader prognostic importance of physiological impairment observed in our cohort. However, our analysis identified ASA class itself as independently associated with mortality, and differences in model composition should be considered when comparing these findings.

Comparative findings for established prognostic tools have varied across studies. Patel et al. reported greater discriminatory performance for PULP than for Boey or ASA [10], whereas a recent meta-analysis identified no significant difference between PULP and Boey in mortality prediction [11]. Since our study did not directly compare these scoring systems, the present results cannot establish superiority of ASA over either PULP or Boey.

An important practical feature of ASA is that it can be determined during preoperative evaluation without requiring additional laboratory testing or calculation of a disease-specific score. However, ASA was not specifically developed for patients with PPU, and its classification may be subject to interobserver variation. Our findings therefore suggest a potential role for ASA as a readily accessible element of early risk assessment, while supporting its use alongside, rather than in place of, established PPU-specific prognostic tools.

Admission BUN levels were higher among non-survivors than survivors (median 36.00 vs. 21.00; *p* < 0.001). However, BUN was not independently associated with in-hospital mortality in the multivariable model (adjusted OR 1.010; 95% CI 0.988–1.032; *p* = 0.378). Thus, the unadjusted group difference and ROC discrimination should not be interpreted as evidence of an independent prognostic effect.

The potential prognostic value of BUN in PPU is supported by previous evidence. Menekse et al. incorporated BUN > 45 mg/dL, together with age and serum albumin, into a practical mortality score [12]. In patients with PPU, however, BUN elevation may represent the combined effects of several acute physiological disturbances rather than renal dysfunction alone. Decreased oral intake, third-space fluid losses, intravascular volume depletion, reduced renal perfusion, and the systemic response to acute illness may all influence BUN concentrations. Thus, BUN may serve as a readily available biochemical marker reflecting the overall physiological burden of acute illness.

Recent evidence from Pouroushaninia et al. highlighted the potential prognostic relevance of BUN in patients undergoing surgery for PPU [15]. In the present cohort, however, the BUN association did not persist after multivariable adjustment. BUN may be influenced by dehydration, renal hypoperfusion, catabolic stress, and underlying renal disease; its prognostic interpretation therefore requires caution.

BUN demonstrated an AUC of 0.749 in our cohort. The ROC-derived threshold of 36.925 mg/dL provided a sensitivity of 53.8% and a specificity of 92.6%, indicating substantially greater specificity than sensitivity. Accordingly, BUN alone would be insufficient for screening or individual clinical decision-making.

The threshold identified in this study was below the BUN value of >45 mg/dL incorporated into the mortality score described by Menekse et al. [12]. However, the 36.925 mg/dL threshold was selected by maximizing the Youden index and was evaluated in the same cohort from which it was derived. Without external validation, this value should be regarded as an exploratory, cohort-derived threshold rather than an established clinical cut-off.

Both BUN and creatinine levels were significantly higher in non-survivors in the nonparametric group comparisons (BUN: median 36.00 vs. 21.00; *p* < 0.001; creatinine: median 1.68 vs. 0.96; *p* < 0.001). Although creatinine is included in the PULP score as an indicator of renal function [8], it was not included in our principal multivariable model. BUN did not remain significant after multivariable adjustment. These findings do not establish either laboratory parameter as an independent predictor in this cohort.

The discriminatory profiles of ASA and BUN differed in this cohort. ASA provided a sensitivity of 92.3%, whereas BUN reached a specificity of 92.6%, suggesting that these parameters may reflect different dimensions of mortality risk. However, their combined prognostic performance was not evaluated. Therefore, the present data do not support any conclusion regarding whether combining ASA with BUN would improve mortality discrimination.

Age differed significantly between groups in the nonparametric comparison (median 76.50 years in non-survivors vs. 55.00 years in survivors; *p* < 0.001). Its ROC AUC was 0.788, with a cohort-derived cut-off of 69.5 years. Age did not remain statistically significant in the principal multivariable model.

Previous cohort studies and systematic reviews have reported an association between advancing age and mortality following PPU [4,5,8,9,17]. In a systematic review of 50 prognostic studies involving 29,782 patients, Møller et al. identified older age and comorbidity as important preoperative mortality-associated factors [5]. Similarly, COPD, hypertension, CAD, DM, CKD, and malignancy were more frequent among non-survivors in our cohort. However, neither age nor the individual comorbidities were independently associated with mortality in the principal multivariable model. These results should be interpreted cautiously in view of limited statistical power and potential overfitting.

Perforation diameter was significantly greater among non-survivors in our cohort. Recent studies have also considered perforation size as a potential marker of disease severity [16,18]. Nevertheless, the independent prognostic contribution of perforation diameter remains uncertain across differing patient populations and statistical models. Because perforation diameter was not included in our principal multivariable model, our results cannot establish it as an independent predictor of mortality.

Median time from symptom onset to hospital admission was 24 h in non-survivors and 16 h in survivors; however, the difference was not statistically significant in the nonparametric comparison (*p* = 0.471). Previous studies have associated treatment delays with adverse outcomes following PPU [5,7,17,19,20], but our results did not demonstrate a significant between-group difference in symptom-to-admission time.

Comparison with previous studies requires caution because the interval measured here extended from symptom onset to hospital admission, rather than from admission to operative source control. Furthermore, symptom-to-admission time was not included in the principal multivariable model. Our data therefore do not establish an independent or causal relationship between presentation delay and mortality.

Hemoglobin levels were lower in non-survivors, although its discriminatory performance was limited, with an AUC of 0.685. Lower hemoglobin in patients with PPU may be influenced by several factors, including underlying comorbidity, gastrointestinal blood loss, nutritional impairment, and reduced physiological reserve. However, its discrimination was weaker than that observed for ASA, age, and BUN, limiting its value as a standalone marker of mortality in the present cohort (Figure 2).

CRP levels were significantly higher among non-survivors than survivors in the nonparametric group comparison (median 11.70 vs. 3.95; *p* = 0.037). CRP also showed modest discriminatory ability in ROC analysis (AUC 0.707; 95% CI 0.548–0.865; *p* = 0.036). However, CRP was not included in the principal multivariable model, and its independent association with mortality was not assessed. Interpretation is further limited by the smaller number of patients with available CRP measurements (*n* = 120).

Recent evidence has also highlighted the prognostic potential of inflammatory and biochemical markers in PPU. In a 2026 retrospective study involving 154 surgically treated patients, Canlikarakaya et al. evaluated CRP and albumin-to-creatinine ratio in relation to 30-day mortality [18]. In our cohort, CRP levels were significantly higher in non-survivors than in survivors (*p* = 0.037) and demonstrated modest discrimination in ROC analysis (AUC = 0.707; 95% CI: 0.548–0.865; *p* = 0.036). Differences in measurement timing, laboratory units, study populations, and statistical adjustment may partly account for these findings. Furthermore, our albumin measurements were obtained postoperatively, preventing direct comparison with preoperative biomarker-based risk assessment.

Postoperative albumin was lower in non-survivors, and when evaluated with ASA in a separate regression model, higher postoperative albumin was associated with lower odds of in-hospital mortality. Previous studies have also linked hypoalbuminemia with unfavorable outcomes in PPU [5,9,12].

However, the postoperative timing of albumin measurement limits its interpretation as an early prognostic marker. In this setting, albumin concentrations may be affected by the physiological response to surgery, systemic inflammation, fluid administration, and altered capillary permeability. Therefore, postoperative albumin is more appropriately interpreted as a marker related to the subsequent clinical course than as a predictor available for risk assessment at admission. Accordingly, Table 5 is a secondary exploratory postoperative prognostic analysis and should not be interpreted as a preoperative risk prediction model.

Postoperative complications, including the requirement for mechanical ventilation, atelectasis, pneumonia, repair-site leakage, and reoperation, were more frequent among non-survivors. These postoperative events should not be interpreted as predictors available at admission, and their association with mortality does not establish causality.

Evidence from emergency surgical populations provides broader context for perioperative risk assessment [21]. A study of the preoperative blood urea nitrogen-to-albumin ratio in acute type A aortic dissection also reported an association with in-hospital mortality [22]; however, its different disease setting and use of a ratio rather than BUN alone preclude direct extrapolation to perforated peptic ulcer. A practical aspect of this study is its emphasis on information already available during routine emergency assessment rather than on the development of another complex prognostic score. ASA class is determined during preoperative evaluation, while BUN can be obtained from routine biochemical testing. However, the findings do not establish the clinical utility of either parameter as a standalone risk prediction tool.

Higher ASA class was associated with mortality after adjustment, whereas BUN differed between mortality groups in the unadjusted comparison and showed discrimination in ROC analysis. Neither parameter should be used alone to guide clinical decisions on the basis of this study, and the prognostic performance of a combined ASA–BUN model was not evaluated.

The findings of this study need to be interpreted within several methodological constraints. Its retrospective design carries inherent risks of selection and information bias, and missing observations were present for some variables. Because missing data were not imputed, analyses were restricted to cases with complete information for the variables being evaluated. Formal normality testing was not documented in the available statistical analysis output; therefore, the suitability of parametric tests for all continuous variables cannot be confirmed. The multivariable model included 13 covariates but only 40 deaths among 265 complete cases (approximately 3.1 events per variable), creating a substantial risk of overfitting and unstable estimates. Adjusted associations should therefore be regarded as exploratory rather than definitive.

The single-center setting represents an additional limitation, as differences in patient characteristics, referral patterns, and clinical practice across institutions may affect the generalizability of our findings.

Calendar year was included in the multivariable model and was not significantly associated with mortality (adjusted OR: 1.032; 95% CI: 0.954–1.117; *p* = 0.431); however, this does not exclude nonlinear temporal effects or differences between treatment periods. A separate period-stratified mortality analysis was not performed.

The long study period also requires consideration. Over the 27 years covered by this cohort, diagnostic imaging, surgical techniques, antimicrobial treatment, resuscitation strategies, anesthetic practice, and intensive care management may have changed considerably. Although calendar year was included in the multivariable model, residual confounding related to temporal changes in care cannot be excluded.

The multivariable analysis was restricted to 265 patients with complete data, including 40 deaths, which may have introduced selection bias and reduced statistical power. Moreover, 13 explanatory variables were included relative to 40 mortality events, increasing the risk of overfitting and unstable estimates. Although calendar year was included as a covariate, period-specific mortality trends and nonlinear temporal effects were not formally evaluated. These limitations should be considered when interpreting the adjusted associations. Missingness stratified by calendar period and survival status could not be reliably characterized from available historical records.

Another limitation concerns the timing of postoperative variables. Postoperative albumin, mechanical ventilation, and time to oral intake reflect aspects of the subsequent clinical course and should not be interpreted as admission-based prognostic indicators. These variables were excluded from the updated admission-based ROC analysis.

In addition, the study was not designed to develop a new mortality prediction model or to directly compare established PPU-specific systems such as PULP, Boey, and MPI. Consequently, our data cannot determine whether adding ASA and BUN to existing risk scores would improve their discriminatory performance or clinical usefulness.

Finally, the ROC thresholds were derived and assessed within the same study population using the Youden index. As these cut-off values have not been tested in an independent population, they remain exploratory and cohort-specific and should not be regarded as validated thresholds for clinical decision-making. PCT availability was time-dependent because testing began in 2016; its period-specific distribution and potential historical changes in assay platform could not be reliably verified from available records, introducing potential selection bias. No combined ASA–BUN ROC model or internal/external validation was performed.

The study includes a substantial cohort of surgically treated patients with PPU accumulated over 27 years of routine clinical practice. Evaluation of demographic, clinical, laboratory, perioperative, and postoperative data within the same cohort provided a broad descriptive assessment of mortality-associated factors. Multivariable regression and ROC analysis were used to examine adjusted associations and discrimination separately; however, the findings remain exploratory and require independent validation.

## 5. Conclusions

In this 27-year retrospective cohort of patients undergoing emergency surgery for PPU, in-hospital mortality was 12.7%. Higher ASA class remained associated with mortality after multivariable adjustment, whereas BUN did not. Older age, lower hemoglobin, higher BUN and creatinine levels, higher CRP, and larger perforation diameter were observed among non-survivors in unadjusted comparisons. Symptom-to-admission time was not significantly different between groups. These findings should be interpreted cautiously because the multivariable model included 13 covariates and only 40 deaths among 265 complete cases.

The study did not directly compare established prognostic scores or assess a combined ASA–BUN model. It therefore does not establish a new or validated prognostic tool.

These findings suggest that ASA classification may contribute to early clinical risk assessment. However, the retrospective single-center design, missing data, and limited number of mortality events in the multivariable analysis warrant cautious interpretation. Further research is needed to validate these findings. ROC-derived thresholds are cohort-specific and require independent validation in prospective multicenter studies.

## Figures and Tables

**Table 1:** Comparison of demographic characteristics and comorbidities according to in-hospital mortality.

Variable	Survivors (*n* = 336)	Non-Survivors (*n* = 49)	*p*-Value
Age, years	55.00 (36.25–69.00)	76.50 (63.25–85.00)	**<0.001**
**Sex, *n* (%)**			**<0.001**
Male	282 (83.9)	31 (63.3)	
Female	54 (16.1)	18 (36.7)	
COPD, *n* (%)	14 (4.2)	9 (18.4)	**<0.001**
Hypertension, *n* (%)	37 (11.0)	18 (36.7)	**<0.001**
Coronary artery disease, *n* (%)	16 (4.8)	12 (24.5)	**<0.001**
Diabetes mellitus, *n* (%)	12 (3.6)	7 (14.3)	**0.001**
Chronic kidney disease, *n* (%)	8 (2.4)	8 (16.3)	**<0.001**
Malignancy, *n* (%)	15 (4.5)	11 (22.4)	**<0.001**

COPD, chronic obstructive pulmonary disease.

**Table 2:** Comparison of clinical and laboratory characteristics according to in-hospital mortality.

Variable	Survivors (*n* = 336)	Non-Survivors (*n* = 49)	*p*-Value
Symptom-to-admission time, h	16.00 (6.00–24.00)	24.00 (0.00–48.00)	0.471
Systolic blood pressure, mmHg	125.00 (115.00–135.00)	120.00 (106.25–130)	0.049
Diastolic blood pressure, mmHg	76.00 (69.00–85.00)	75.00 (60.00–80.00)	0.138
Heart rate, beats/min	86.00 (76.00–99.25)	85.50 (76.75–100.75)	0.998
White blood cell count	13.40 (9.20–17.10)	10.60 (3.80–16.70)	**0.024**
Hemoglobin	14.90 (13.60–16.20)	12.30 (10.40–14.18)	**<0.001**
BUN	21.00 (15.00–31.45)	36.00 (23.00–53.00)	**<0.001**
Creatinine	0.96 (0.80–1.30)	1.68 (1.10–2.29)	**<0.001**
Procalcitonin	2.96 (0.74–21.00)	14.60 (2.45–35.5)	0.202
CRP	3.95 (0.99–12.25)	11.70 (3.82–13.65)	**0.037**
Perforation diameter, cm	0.50 (0.50–1.00)	1.00 (0.50–1.25)	**<0.001**

BUN, blood urea nitrogen; CRP, C-reactive protein.

**Table 3:** Comparison of perioperative and postoperative characteristics according to in-hospital mortality.

Variable	Survivors (*n* = 336)	Non-Survivors (*n* = 49)	*p*-Value
Postoperative albumin	2.80 (2.40–3.30)	2.00 (1.60–2.60)	**<0.001**
Duration of anesthesia, min	90.00 (60.00–120.00)	120.00 (120.00–130.00)	**<0.001**
Time to drain removal, days	5.00 (4.00–6.00)	0.00 (0.00–8.00)	**<0.001**
Time to nasogastric tube removal, days	3.00 (2.00–4.00)	6.00 (3.00–9.50)	**<0.001**
Time to initiation of oral feeding, days	4.00 (3.00–5.00)	7.00 (3.75–9.50)	**<0.001**
Length of hospital stay, days	6.00 (5.00–9.00)	7.00 (3.00–14.00)	0.482
Duration of mechanical ventilation, days	1.00 (0.00–2.00)	5.50 (3.00–9.50)	**<0.001**
Mechanical ventilation, *n* (%)	29 (8.6)	38 (77.6)	**<0.001**
Atelectasis, *n* (%)	25 (7.4)	16 (32.7)	**<0.001**
Pneumonia, *n* (%)	20 (6.0)	19 (38.8)	**<0.001**
Perforation repair-site leakage, *n* (%)	7 (2.1)	5 (10.2)	**0.002**
Reoperation, *n* (%)	4 (1.2)	3 (6.1)	**0.016**
Surgical site infection, *n* (%)	28 (8.3)	2 (4.1)	0.300
Evisceration, *n* (%)	14 (4.2)	2 (4.1)	0.978
Postoperative ileus, *n* (%)	9 (2.7)	2 (4.1)	0.582

**Table 4:** Multivariable logistic regression analysis of factors associated with in-hospital mortality (*n* = 265; 40 deaths).

Variable	B	SE	Adjusted OR	95% CI	*p*-Value	VIF
ASA class	1.687	0.366	5.404	2.640–11.064	<0.001	2.088
Male sex	0.912	0.506	2.489	0.923–6.713	0.072	1.113
Age	0.003	0.015	1.003	0.973–1.034	0.857	1.944
COPD	0.678	0.734	1.970	0.467–8.308	0.356	1.172
Hypertension	−0.086	0.519	0.917	0.331–2.538	0.868	1.476
Coronary artery disease	0.642	0.621	1.900	0.563–6.418	0.301	1.173
Diabetes mellitus	0.277	0.708	1.319	0.329–5.286	0.696	1.131
Chronic kidney disease	1.253	0.767	3.500	0.779–15.732	0.102	1.279
Malignancy	0.948	0.638	2.580	0.738–9.012	0.138	1.182
Systolic blood pressure	−0.010	0.017	0.990	0.958–1.024	0.564	2.001
Diastolic blood pressure	0.016	0.024	1.016	0.969–1.065	0.511	1.874
BUN	0.010	0.011	1.010	0.988–1.032	0.378	1.544
Calendar year	0.032	0.040	1.032	0.954–1.117	0.431	1.271

Abbreviations: B, regression coefficient; SE, standard error; OR, odds ratio; CI, confidence interval; VIF, variance inflation factor; COPD, chronic obstructive pulmonary disease; BUN, blood urea nitrogen. All variables were entered simultaneously using the Enter method.

**Table 5:** Logistic regression analysis of the association of ASA class and postoperative albumin with in-hospital mortality.

Variable	B	SE	OR	95% CI	*p*-Value
ASA class	1.616	0.366	5.032	2.454–10.319	<0.001
Postoperative albumin	−0.812	0.389	0.444	0.207–0.952	0.037
Constant	−3.610	1.581	0.027	—	0.022

ASA, American Society of Anesthesiologists; CI, confidence interval; OR, odds ratio; SE, standard error.

**Table 6:** Receiver operating characteristic (ROC) analysis of clinical and laboratory variables for predicting in-hospital mortality.

Variable	*n*	Cutoff	Sensitivity	Specificity	AUC	SE	*p*-Value	95% CI
Age, years	372	69.500	0.769	0.778	0.788	0.079	0.004	0.633–0.942
ASA class	296	2.500	0.923	0.704	0.801	0.071	0.002	0.661–0.941
CRP	120	2.775	1.000	0.444	0.707	0.081	0.036	0.548–0.865
BUN	343	36.925	0.538	0.926	0.749	0.086	0.012	0.581–0.917

Abbreviations: AUC, area under the receiver operating characteristic curve; ASA, American Society of Anesthesiologists physical status classification; BUN, blood urea nitrogen; CI, confidence interval; CRP, C-reactive protein; ROC, receiver operating characteristic; SE, standard error.

**Table 7:** Receiver operating characteristic (ROC) analysis of hemoglobin level for predicting in-hospital mortality.

Variable	*n*	Cutoff	Sensitivity	Specificity	AUC	SE	*p*-Value	95% CI
Hemoglobin	349	14.250	0.795	0.617	0.685	0.053	<0.001	0.582–0.788

Abbreviations: AUC, area under the receiver operating characteristic curve; CI, confidence interval; ROC, receiver operating characteristic; SE, standard error.

## Data Availability

The data supporting the findings of this study are available from the corresponding author upon reasonable request, subject to institutional approval and applicable patient confidentiality restrictions.

## References

[1] Søreide K., Thorsen K., Harrison E.M., Bingener J., Møller M.H., Ohene-Yeboah M., Søreide J.A. (2015). Perforated peptic ulcer. Lancet.

[2] Søreide K., Thorsen K., Søreide J.A. (2014). Strategies to improve the outcome of emergency surgery for perforated peptic ulcer. Br. J. Surg..

[3] Tarasconi A., Coccolini F., Biffl W.L., Tomasoni M., Ansaloni L., Picetti E., Molfino S., Shelat V., Cimbanassi S., Weber D.G. (2020). Perforated and bleeding peptic ulcer: WSES guidelines. World J. Emerg. Surg..

[4] Thorsen K., Søreide J.A., Kvaløy J.T., Glomsaker T., Søreide K. (2013). Epidemiology of perforated peptic ulcer: Age- and gender-adjusted analysis of incidence and mortality. World J. Gastroenterol..

[5] Møller M.H., Adamsen S., Thomsen R.W., Møller A.M. (2010). Preoperative prognostic factors for mortality in peptic ulcer perforation: A systematic review. Scand. J. Gastroenterol..

[6] Thorsen K., Søreide J.A., Søreide K. (2013). Scoring systems for outcome prediction in patients with perforated peptic ulcer. Scand. J. Trauma Resusc. Emerg. Med..

[7] Boey J., Choi S.K., Poon A., Alagaratnam T.T. (1987). Risk stratification in perforated duodenal ulcers: A prospective validation of predictive factors. Ann. Surg..

[8] Møller M.H., Engebjerg M.C., Adamsen S., Bendix J., Thomsen R.W. (2012). The Peptic Ulcer Perforation (PULP) score: A predictor of mortality following peptic ulcer perforation. A cohort study. Acta Anaesthesiol. Scand..

[9] Thorsen K., Søreide J.A., Søreide K. (2014). What is the best predictor of mortality in perforated peptic ulcer disease? A population-based, multivariable regression analysis including three clinical scoring systems. J. Gastrointest. Surg..

[10] Patel S., Kalra D., Kacheriwala S., Shah M., Duttaroy D. (2019). Validation of prognostic scoring systems for predicting 30-day mortality in perforated peptic ulcer disease. Turk. J. Surg..

[11] Christanto A.G.R., Adrianto A.A. (2026). Comparative accuracy of Boey score and PULP score for predicting prognosis in patients with perforated peptic ulcer: A systematic review and meta-analysis. J. Visc. Surg..

[12] Menekse E., Kocer B., Topcu R., Olmez A., Tez M., Kayaalp C. (2015). A practical scoring system to predict mortality in patients with perforated peptic ulcer. World J. Emerg. Surg..

[13] De Cassai A., Boscolo A., Tonetti T., Ban I., Ori C. (2019). Assignment of ASA-physical status relates to anesthesiologists’ experience: A survey-based national-study. Korean J. Anesthesiol..

[14] Yalcin M., Oter S., Akinoglu A. (2022). Early post-operative morbidity and mortality predictors in peptic ulcer perforation. Ulus. Travma Acil Cerrahi Derg..

[15] Pouroushaninia N., Bozorgmehr R., Alibeik N., Ahmadinezhad M., Bagherpour J.Z., Rashidian M., Bahri M.H. (2025). Surgical Mortality and Morbidity Predictors in Patients With Perforated Peptic Ulcer: A Cohort Study. J. Surg. Res..

[16] Hoshi H., Endo A., Ito K., Akutsu T., Odera H., Shiraki H., Ito K., Yokoyama T., Narita Y., Masuda T. (2025). Analysis of the characteristics and management of perforated peptic ulcer from 2011 to 2022: A multicenter and retrospective descriptive study. Ann. Gastroenterol. Surg..

[17] Kocer B., Surmeli S., Solak C., Unal B., Bozkurt B., Yildirim O., Dolapci M., Cengiz O. (2007). Factors affecting mortality and morbidity in patients with peptic ulcer perforation. J. Gastroenterol. Hepatol..

[18] Canlikarakaya F., Ocakli S., Dogan I., Cinkil N.C., Ozmen S.N., Ceylan C. (2026). The role of biomarkers in predicting mortality in peptic ulcer perforation. BMC Surg..

[19] Buck D.L., Vester-Andersen M., Møller M.H., Danish Clinical Register of Emergency Surgery (2013). Surgical delay is a critical determinant of survival in perforated peptic ulcer. Br. J. Surg..

[20] Boyd-Carson H., Doleman B., Cromwell D., Lockwood S., Williams J.P., Tierney G.M., Lund J.N., Anderson I.D., National Emergency Laparotomy Audit Collaboration (2020). Delay in source control in perforated peptic ulcer leads to 6% increased risk of death per hour: A nationwide cohort study. World J. Surg..

[21] Merani S., Payne J., Padwal R.S., Hudson D., Widder S.L., Khadaroo R.G. (2014). Predictors of in-hospital mortality and complications in very elderly patients undergoing emergency surgery. World J. Emerg. Surg..

[22] Wu Q., Zheng J., Lin J., Xie L., Tang M., Ke M., Chen L. (2024). Preoperative blood urea nitrogen-to-serum albumin ratio for prediction of in-hospital mortality in patients who underwent emergency surgery for acute type A aortic dissection. Hypertens. Res..

